# Active Packaging Film Developed by Incorporating Starch Aldehyde–Quercetin Conjugate into SPI Matrix

**DOI:** 10.3390/antiox13070810

**Published:** 2024-07-04

**Authors:** Yufeng Sun, Yang Ju, Qinfei Xie, Ran Tao, Lili Wang, Bei Fan, Fengzhong Wang

**Affiliations:** Institute of Food Science and Technology, Chinese Academy of Agricultural Sciences, Beijing 100193, China; yufengsuncaas@163.com (Y.S.); j2596997473@163.com (Y.J.); 19113526360@163.com (Q.X.); ran.tao5@mail.mcgill.ca (R.T.); wlland2013@163.com (L.W.)

**Keywords:** soy protein isolate, dialdehyde starch, quercetin, packaging, fresh cut, preservation

## Abstract

In this study, soy protein isolate (SPI) films incorporating quercetin-grafted dialdehyde starch (DAS-QR) and DAS/QR, respectively, were developed. The structural, physical, and functional properties of the composite films were determined. The results suggested that DAS-QR and DAS/QR formed hydrogen bonding with the SPI matrix, which improved the structural properties of the films. The light-blocking capacity, thermal stability, hydrophobicity, tensile strength, elongation at break, and antioxidant and antibacterial abilities of SPI films were improved by DAS-QR and DAS/QR. Notably, SPI films incorporated with DAS-QR exhibited better performance than those with DAS/QR in terms of antioxidant (SPI/DAS-QR: 79.8% of DPPH and 62.1% of ABTS scavenging activity; SPI/DAS/QR: 71.4% of DPPH and 56.0% of ABTS scavenging activity) and antibacterial abilities against *S. aureus* (inhibition rate: 92.7% for SPI/DAS-QR, 83.4% for SPI/DAS/QR). The composite coating film SPI/DAS-QR effectively maintained appearance quality, delayed the loss of weight and total soluble solids, postponed malondialdehyde accumulation, and decreased peroxidase activity and microbial contamination in fresh-cut potatoes. These good performances highlight SPI/DAS-QR as a promising active packaging material for fresh-cut product preservation.

## 1. Introduction

With the public’s growing attention on environmental protection and food safety in recent years, biopolymer-based food packaging has received increasing interest due to its accessibility, biodegradability, and cost-effectiveness [1,2]. Proteins, polysaccharide polysaccharides, and lipids are prevalent biopolymers used for developing food packaging. Biopolymer-based food packaging, incorporating antimicrobial and antioxidant components, can reduce microbial contamination, oxidation, and food spoilage during storage and transportation [3]. Soy protein isolate (SPI) is the primary protein found in soybean meal. SPI is a good choice for producing biodegradable food packaging films due to its excellent film-forming characteristics, abundant availability, and edible nature [4]. Nonetheless, neat SPI films have been demonstrated with unsatisfactory mechanical strength, barrier properties, and hydrophobicity [5]. Incorporating aldehydes such as formaldehyde, glyoxal, and glutaraldehyde can boost cross-linking among protein chains. This enhancement contributes to improving the physical properties of protein films [6]. Dialdehyde starch (DAS) is an aldehyde obtained by oxidizing starch with sodium periodate. It has been applied to improve film properties [7]. Wang et al. prepared a gelatin film containing 10% DAS that showed enhanced tensile strength (TS), hydrophobicity, thermal stability, and UV-blocking properties [8]. Meanwhile, neat SPI films have been demonstrated with unsatisfactory functional activity. Therefore, they are frequently utilized in combination with natural bioactive substances to develop packaging materials. Yu et al. demonstrated that SPI film containing peppercorn leaf extract exhibited enhanced antioxidant activity, which extended the shelf life of cherry tomatoes [9].

Phenolic compounds are known for their antioxidant properties, which are attributed to the efficient quenching of free radicals by phenolic hydroxyl groups [10]. Moreover, they can cause damage to the cell wall of microorganisms, increase the permeability of the cell membrane, and thus exhibit antimicrobial activity [11]. In recent years, many researchers have fabricated active food packaging materials by the combination of phenolic compounds and a biopolymer matrix. Song et al. fabricated chitosan (CS) film containing magnolol, which exhibited enhanced antioxidant activity and good efficacy in pork preservation [12]. Tao et al. found that SPI film incorporating carvacrol exhibited antibacterial activity [13].

Quercetin (QR) is a polyphenolic compound widely existing in plants. It can scavenge oxygen free radicals responsible for initiating lipid peroxidation [14,15]. QR is frequently incorporated into films to impart antioxidant and antimicrobial properties, thereby preserving food quality, slowing down spoilage, and prolonging shelf life [14]. Jakubowska et al. found that the addition of QR caused an increase in antioxidant activity in native CS film and successfully delayed secondary lipid oxidation processes [16]. Rani et al. found that the introduction of QR could enhance the antibacterial activity against *Listeria monocytogenes* (*L. monocytogenes*) and *Escherichia coli* (*E. coli*) in SPI film [17].

The application forms of natural bioactive substances in bio-based films include (1) direct addition, and (2) grafting with biopolymers to form conjugates, and then adding them. Both of these methods can improve the performance of films in terms of mechanical properties, and antioxidant and antimicrobial activities [18]. Some researchers have demonstrated that packaging films prepared by the latter method are more effective than those produced by the former method [19,20]. In recent years, DAS–polyphenol conjugates have displayed promising applications in food packaging. The incorporation of DAS–catechin conjugate into quaternary ammonium chitosan/poly (vinyl alcohol) could improve homogeneity, mechanical properties, and gas- and vapor-blocking capacities [21]. Chitosan in combination with DAS–catechin conjugate could effectively delay the oxidation and spoilage of pork loins during chilled storage [22]. However, few studies have reported whether the use of DAS–polyphenol conjugates in packaging films is more effective than directly adding DAS and polyphenols.

**Figure 1:**
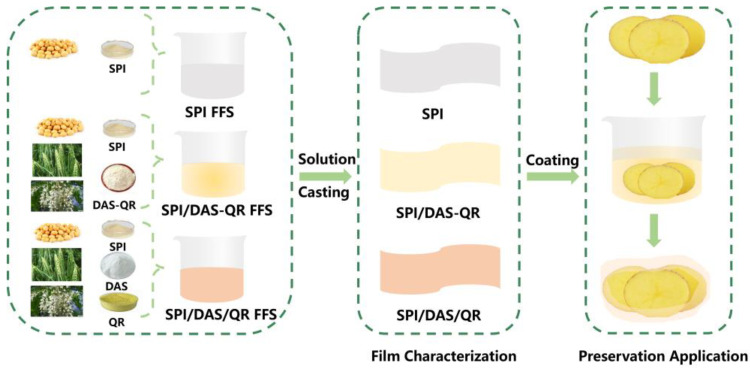
Diagram of the synthesis and application of the films.

In this study, a novel active packaging film, created by incorporating DAS-QR conjugate into an SPI film matrix, as well as another film created by directly adding DAS and QR, were developed (Figure 1). The developed films were characterized in terms of structural, physical, antioxidant, and antibacterial properties. Finally, the application potential of the films in fresh-cut potato packaging was evaluated.

## 2. Materials and Methods

### 2.1. Materials

SPI, QR, sodium periodate, choline chloride, and 2, 2-diphenyl-1-picrylhydrazyl (DPPH) were obtained from Macklin (Shanghai, China). *Staphylococcus aureus* (*S. aureus*) and *E. coli* were purchased from Huankai (Guangzhou, China). A 2, 2′-azino-bis(3-ethylbenzothiazoline-6-sulfonic acid) (ABTS) free-radical scavenging ability test kit was obtained from Solarbio (Beijing, China). Malondialdehyde (MDA) and peroxidase (POD) activity test kits were obtained from Grace (Beijing, China). All other reagents were of analytical grade and purchased from Yuanye (Shanghai, China). Starch from Qingke (Tibetan barley) was extracted as described previously [23]. Potatoes (*Solanum tuberosum* L.) were collected from a local farmers market (Beijing, China). The potatoes used for the treatment exhibited a consistent size, shape, and color, and were devoid of any impairment or deterioration.

### 2.2. Preparation of Qingke DAS-QR Conjugate

Qingke DAS-QR conjugate was prepared with an acid-catalyzed condensation reaction, as described previously [24]. Briefly, 3.25 g of sodium periodate was dissolved in 25 mL of distilled water, followed by 2.5 g of Qingke starch. The pH of the mixture was adjusted to 3.5. The reaction was conducted at 40 °C for 4.5 h in the dark. The product was washed, dialyzed, and dried to afford Qingke DAS. Then, 0.25 g of Qingke DAS was dissolved in 6.5 mL of 1 M HCl solution. Meanwhile, 1.25 g of QR was dissolved in 15 mL of dimethyl sulfoxide. Next, the two solutions were mixed together and reacted at 45 °C for 48 h in the dark. Finally, the product was dialyzed and lyophilized to give Qingke DAS-QR conjugate. The conjugation efficiency was determined to be 58.98 mg/g by colorimetric methods.

### 2.3. Development of the Films

First, deep eutectic solvent (DES) was prepared according to previous research [25]. Briefly, 4.64 g of choline chloride and 2.0 g of urea were dissolved in 1.0 mL of distilled water. The mixture was heated to 80 °C and stirred until it formed a clear liquid to afford DES. The films were prepared by the solution casting method [9]. Specifically, 6.0 g of SPI was dissolved in 100 mL of distilled water and stirred at 80 °C for 1 h. The pH of the solution was adjusted to 8 with sodium hydroxide solution (1 mol/L), followed by the addition of DES (1.5 g). Then, 0.3 g of DAS, 0.24 g of QR, and 0.3 g of Qingke DAS-QR conjugate were added to the SPI solution, followed by continuous stirring at 25 °C for 30 min and sonication at 400 W for 30 min. The film-forming solution (FFS) was poured into molds and dried at 55 °C for 4 h. The SPI films containing DAS/QR and DAS-QR were named SPI/DAS/QR and SPI/DAS-QR, respectively. All the films were placed in a desiccator at 25 °C with 50% relative humidity (RH) for 48 h.

### 2.4. Structural Properties of Films

The internal morphologies of the films were observed using SEM SU8100 (Hitachi, Tokyo, Japan) at a voltage of 5 kV. The FT-IR spectrum of the films was defined by a TENSOR FTIR spectrometer (Bruker, SB, Ettlingen, Germany) from 4000 to 500 cm^−1^. The crystallinity of the films was investigated with an Ultima IV X-ray diffractometer (Rigaku, Tokyo, Japan) at 10°–60°.

### 2.5. Physical Properties of the Films

An electronic eye DigiEye System (Verivide, Leicester, UK) with a standard light source D65 was used for investigating the color of the films. The total color difference (ΔE) was calculated according to the following equation [26]:

(1) $$\Delta E=\sqrt{{\left(L-L_{0}\right)}^{2}+{\left(a-a_{0}\right)}^{2}+{\left(b-b_{0}\right)}^{2}}$$

where L_0_, a_0_, and b_0_ are the standard values of a white plate, and L, a, and b are the measured color profile values of the film samples. The standard values of the white calibration plate were L_0_ = 97.39, a_0_ = 0.03, and b_0_ = 1.77.

The light transmittance of the films was measured by a UV–Vis spectrophotometer UV8000s (Yuanxi, Shanghai, China) at 200–600 nm.

Thermal gravimetric analysis (TGA) was used for evaluating the thermal properties of the films, and was performed on the Pyris Diamond TGA instrument (Perkin Elmer, Waltham, MA, USA). The film samples (3 mg) were heated from 30 °C to 500 °C at 10 °C/min in a N^2^ atmosphere. The flow rate of N^2^ was 20 mL/min.

The water contact angle (WCA) of the films was determined using OCA25 (DataPhysics, Filderstadt, Germany). The volume of the sample was 2 μL.

The mechanical properties of the films, including tensile strength (TS) and elongation at break (EB), were investigated according to previous research [27]. Briefly, a film strip (1 × 5 cm) was placed on a TA.HD plus physical property tester (Stable Micro Systems, Surrey, UK) and stretched at a speed of 1 mm/s. The TS and EB were calculated as follows:

(2) $$TS=\frac{F}{S}$$

(3) $$EB\left(\%\right)=\frac{\Delta L}{L_{0}}\times 100$$

where F (N) represents the maximum force, S (mm^2^) represents the cross-sectional area of the film, ΔL (mm) is the length change of the film post-deformation, and L_0_ (mm) is the original marking distance of the film.

### 2.6. Antioxidant Properties of the Films

The antioxidant activity of the films was expressed by DPPH and ABTS radical scavenging activity, and measured according to the previous method, with some modifications [28]. Briefly, film strips (2 × 2 cm) were immersed in 3 mL of DPPH methanol solution (0.1 mmol/L) in the dark at 20 °C for 30 min. After centrifugation at 8000 rpm for 5 min, the absorbance of the solution was recorded at 517 nm. The DPPH radical scavenging activity was calculated via Formula (4). Film strips (2 × 2 cm) were immersed in 3 mL of methanol in the dark at 20 °C for 30 min. The solution was used to determine the ABTS radical scavenging activity according to the kit instructions. The absorbance at 405 nm was measured and the ABTS radical scavenging activity was calculated via Formula (5).

(4) $$DPPH\;radical\;scavenging\;activity\left(\%\right)=\frac{A_{1}-A_{2}}{A_{1}}\times 100$$

where A_1_ represents the absorbance of the reference (DPPH methanol solution) and A_2_ represents the absorbance of the test sample.

(5) $$ABTS\;radical\;scavenging\;activity\left(\%\right)=\frac{{A_{0}-(A}_{t}-A_{c})}{A_{0}}\times 100$$

where A_0_ represents the absorbance of the blank (distilled water), A_t_ represents the absorbance of the test sample under test-treated conditions, and A_c_ represents the absorbance of the test sample under control-treated conditions.

### 2.7. Antibacterial Properties of the Films

The antibacterial activity of the films was evaluated by referring to the method described in previous research with slight modifications [29]. The tested bacteria were Gram-positive bacteria *S. aureus* and Gram-negative bacteria *E. coli*. An amount of 9 mL of bacteria suspension (10^5^ CFU/mL) was mixed with 1 mL of film-forming solution, and incubated at 37 °C for 2 h. Afterwards, the mixture was diluted tenfold. Then, 150 μL of the diluted mixture was inoculated onto an agarose Petri dish (*Φ*: 9 cm), which was then incubated at 37 °C for 18 h. Finally, the bacterial count was determined.

### 2.8. Application of Coating Films for Fresh-cut Potato Preservation

Potatoes with a consistent size (*Φ*: 7 cm), shape, and color were washed, peeled, sliced (T: 1.5 cm), and randomly divided into three groups. The fresh-cut potatoes were immersed in SPI, SPI/DAS/QR, and SPI/DAS-QR film-forming solutions for 2 min, respectively. After air-drying until there was no solvent on the surface, the fresh-cut potatoes were stored (4 °C) for 12 d. During the storage, the quality of the potatoes was assessed every 3 d in terms of color, weight loss, total soluble solids (TSS), MDA content, POD activity, and total colony count.

The color of the fresh-cut potatoes was investigated by electronic eye with a standard light source D65. ΔE was the index of total color change and was calculated via Formula (1).

The weight loss of the fresh-cut potatoes was expressed by the change in weight every 3 d. It was calculated as follows:

(6) $$W\left(\%\right)=\frac{W_{1}-W_{2}}{W_{1}}\times 100$$

where W_1_ (g) and W_2_ (g) are the initial weight and the weight after every 3 d, respectively.

The fresh-cut potato samples were homogenized for 3 min at 5 Kr/min. The TSS content was subsequently determined using a 2WAJ Abbe Refractometer (Shanghai Optical Instrument, Shanghai, China).

The MDA and POD content of the fresh-cut potatoes were measured with test kits according to the instructions. The results are expressed in mmol·g^−1^.

The total colony count on the fresh-cut potatoes was measured according to previous research [20]. Briefly, the fresh-cut potato samples (1 g) were added to a normal saline solution (10 mL) and homogenized for 3 min at 5 Kr/min. The supernatant (100 μL) was coated on an agar plate (*Φ*: 9 cm), which was incubated at 37 °C for 48 h. Then, the total colony count was recorded.

### 2.9. Statistical Analysis

Statistical analysis was performed by one-way analysis of variance and Duncan’s multiple range test (*p* < 0.05) using SPSS 13.0 software (SPSS Inc., Chicago, IL, USA).

## 3. Results and Discussion

### 3.1. Internal Morphological Properties of the Films

**Figure 2:**
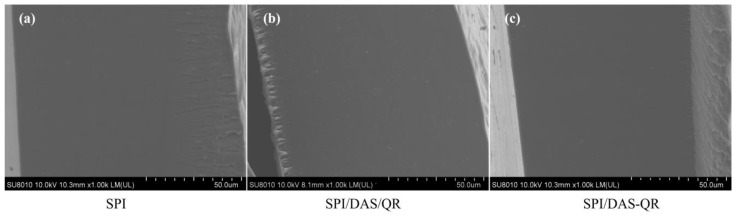
Cross-section SEM images of (**a**) SPI film, (**b**) SPI/DAS/QR film, and (**c**) SPI/DAS-QR film.

SEM is an important tool for investigating the microstructure of films and the compatibility between components [30]. SEM images of the cross-sections of the different films are shown in Figure 2. It can be observed that the neat SPI film was intact, smooth, and uniform without any cracks, revealing the good compatibility of SPI and DES. After DAS and QR were added to the matrix, the cross-section of the SPI/DAS/QR film displayed particles, which may be due to the poor water solubility of QR. It was observed that the cross-section of the SPI/DAS-QR film remained smooth and uniform, suggesting DAS-QR is compatible with the matrix.

### 3.2. FTIR Analysis of the Films

**Figure 3:**
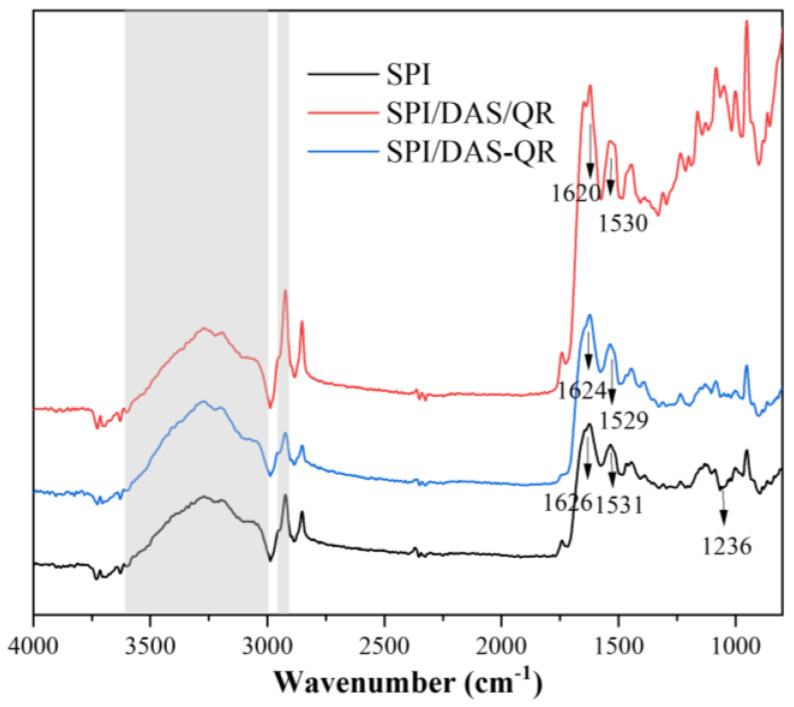
FTIR spectra of the SPI, SPI/DAS/QR, and SPI/DAS-QR films.

The interactions between SPI and the additives were investigated by FTIR spectroscopy. As shown in Figure 3, the FTIR spectrum of the SPI film displayed characteristic absorption peaks at 3600–3000 cm^−1^ and 2915–2935 cm^−1^, corresponding to the O-H stretching of amide A and the N-H stretching of amide B, respectively. There were characteristic absorption peaks at 1626, 1531, and 1236 cm^−1^, assigned to the C=O stretching vibration of amide I, the N-H bending band of amide II, and the C-N stretching of amide III [31]. The composite films SPI/DAS/QR and SPI/DAS-QR exhibited a similar FTIR spectrum to the SPI film. Notably, the intensity of the broad absorption peak at 3600–3000 cm^−1^ for the SPI film slightly decreased when DAS/ QR and DAS-QR were added. Meanwhile, the characteristic absorption peak at 1626 cm^−1^ moved slightly to 1620 and 1624 cm^−1^, and the characteristic absorption peak at 1531 cm^−1^ moved slightly to 1530 and 1529 cm^−1^, respectively. The results revealed the formation of hydrogen bonding between the matrix and the added substances [32]. This interaction enhanced the energy dissipation within the film, contributing to an overall improvement in structural properties [5].

### 3.3. XRD Analysis of the Films

**Figure 4:**
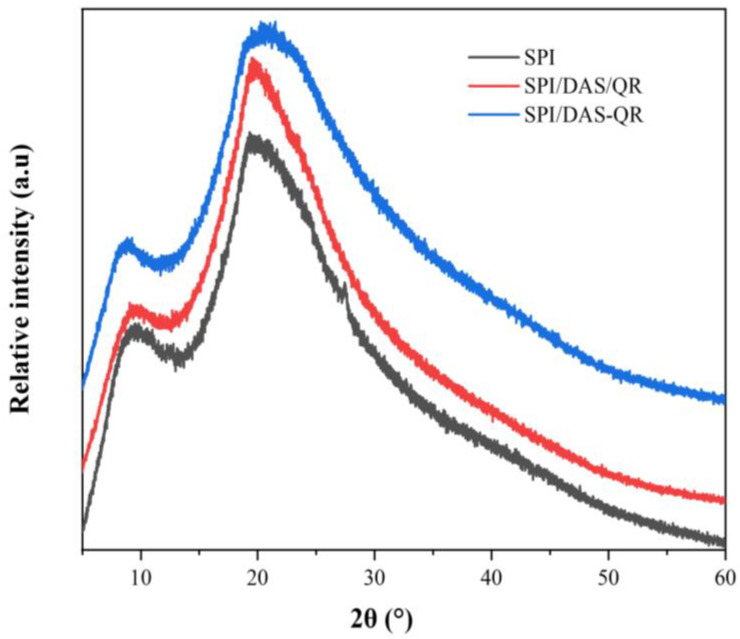
XRD spectra of the SPI, SPI/DAS/QR, and SPI/DAS-QR films.

Crystallinity is a vital index of materials, and can be determined by XRD analysis [32]. As shown in Figure 4, the SPI film exhibited two peaks near the 2*θ* values of 9.5° and 19.8°, corresponding to the α helix and β sheet structure of SPI’s secondary conformation [33]. The composite films SPI/DAS/QR and SPI/DAS-QR exhibited similar XRD spectra to the SPI film. Notably, the intensity of the peaks slightly decreased, indicating a decrease in crystallinity. This may be attributed to the change in the structural order caused by the cross-linking interaction between the phenolic hydroxyl in the QR moiety and the amino group of the SPI through hydrogen bonding [33,34].

### 3.4. Color and Light Transmittance of the Films

**Figure 5:**
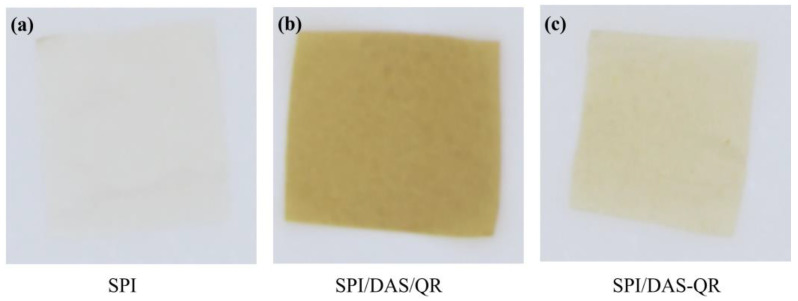
The sample of (**a**) SPI film, (**b**) SPI/DAS/QR film, and (**c**) SPI/DAS-QR film.

The SPI films with and without DAS/QR and DAS-QR are displayed in Figure 5, and the color parameters are summarized in Table 1. The neat SPI film was bright and almost colorless, with a high L and low a, b, and ΔE. The composite films SPI/DAS/QR and SPI/DAS-QR became less bright and more yellow with the decreased L and increased a, b, and ΔE. This was attributed to the color introduced by the QR moiety.

**Figure 6:**
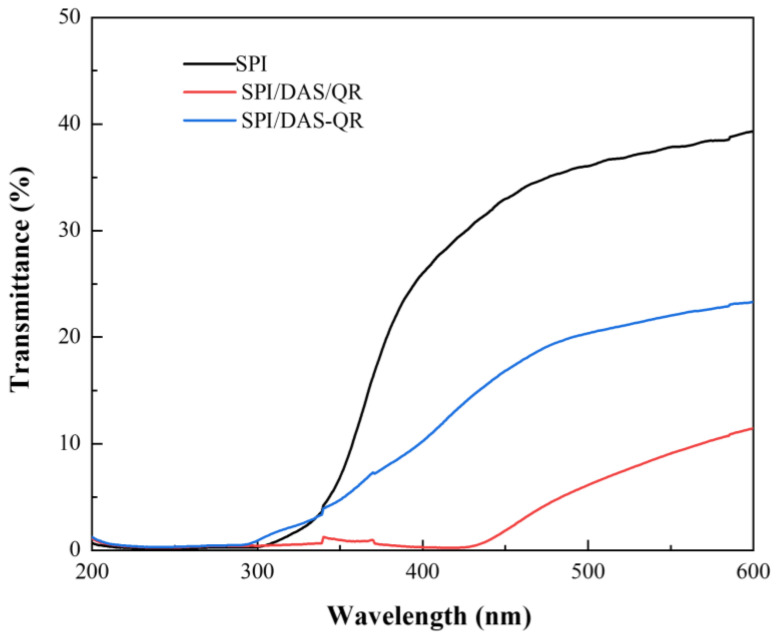
The light transmittance of the SPI, SPI/DAS/QR, and SPI/DAS-QR films.

Light has a negative impact on food quality. Ideal food packaging exhibits high light-blocking capacity. The transmittance of the SPI, SPI/DAS/QR, and SPI/DAS-QR films was evaluated. As shown in Figure 6, SPI/DAS/QR and SPI/DAS-QR displayed lower transmittance compared with the SPI film. The results indicate that the composite films exhibit higher light-blocking capacity than the neat film. This may be due to the colored OR and DAS-QR, which are light absorbers [35]. In addition, the incorporation of DAS/QR and DAS-QR enhanced the densification of the films, which altered the refractive index [36]. Notably, SPI/DAS/QR displayed higher light-blocking capacity than SPI/DAS-QR. This may be due to the higher amount of QR in the SPI/DAS/QR film. However, food packaging that is too dark could affect consumer perceptions.

### 3.5. TGA of the Films

**Figure 7:**
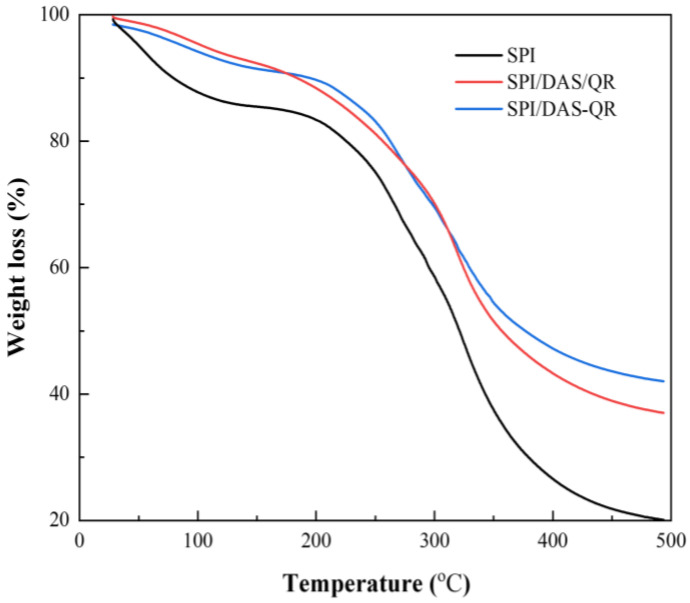
The TGA curves of the SPI, SPI/DAS/QR, and SPI/DAS-QR films.

Thermal stability is an essential property of food packaging that influences its performance during storage [16]. The thermal decomposition behavior of SPI films with and without DAS/QR and DAS-QR are shown in Figure 7. It was observed that the SPI film underwent three distinct phases of weight loss. The first stage occurred from 30 °C to 200 °C, which was due to the evaporation of free-bound water in the film, accompanied by the loss of intramolecular and intermolecular hydrogen bonds. The second stage occurred from 200 °C to 350 °C, which was associated with the decomposition of polymer molecules and the loss of bound water [37]. The third stage took place from 350 °C to 500 °C, which was caused by the combustion of the carbon skeleton. The composite films SPI/DAS/QR and SPI/DAS-QR displayed similar TGA curves with the neat SPI film. However, the SPI/DAS/QR and SPI/DAS-QR films showed slower weight loss rates compared with the SPI film at the three stages. This may be due to their more compact structures caused by the cross-linking of the molecules. The results indicate that the addition of DAS/QR and DAS-QR could increase the thermal stability of SPI film.

### 3.6. WCA of the Films

**Figure 8:**
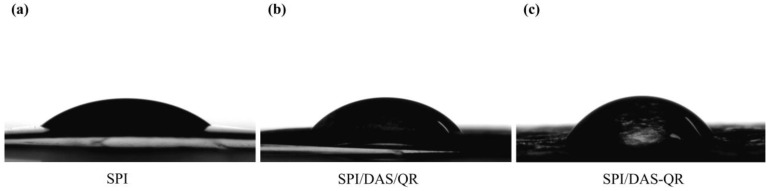
WCA images of (**a**) SPI film, (**b**) SPI/DAS/QR film, and (**c**) SPI/DAS-QR film.

WCA can reflect the hydrophobicity and interfacial wettability of food packaging [38]. As shown in Figure 8, the SPI film exhibited a small WCA (33.10°), suggesting a low hydrophobicity. This was attributed to the interaction of hydroxyl and amino groups with water in the SPI film. The SPI/DAS/QR (42.10°) and SPI/DAS-QR (52.83°) films exhibited bigger WCA, indicating a higher hydrophobicity. This may be due to the consumption of the inherent hydrophilic groups of SPI with the addition of DAS/QR and DAS-QR. In addition, the decrease in free volume in the composite films due to the denser structures led to the enhancement of hydrophobicity.

### 3.7. Mechanical Properties of the Films

**Figure 9:**
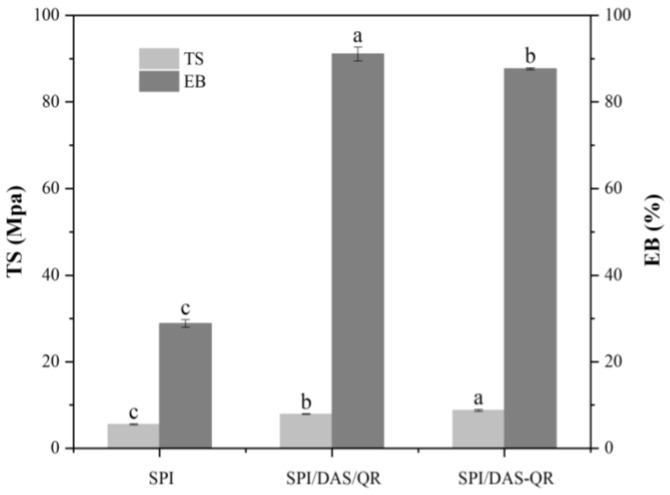
TS and EB of the SPI, SPI/DAS/QR, and SPI/DAS-QR films. a–c: different leters indicate significant differences among the films (*p* < 0.05).

Mechanical properties are an important indicator of food packaging. As shown in Figure 9, the TS of the SPI film was 5.56 MPa, and the EB was 28.85%. The incorporation of DAS/QR and DAS-QR with SPI significantly improved the TS. The TS values of the SPI/DAS/QR and SPI/DAS-QR films were 7.95 and 8.81 MPa, respectively. The decrease in TS was attributed to the increase in rigidity caused by the hydrogen bond interaction between the amino groups of SPI and the hydroxyl groups of the OR moiety. The incorporation of DAS/QR and DAS-QR with SPI slightly improved the EB. The EB values of the SPI/DAS/QR and SPI/DAS-QR films were 91.09% and 87.69%, respectively. This may be due to the slight change in the entanglement of the film structures [9].

### 3.8. Antioxidant Activities of the Films

**Figure 10:**
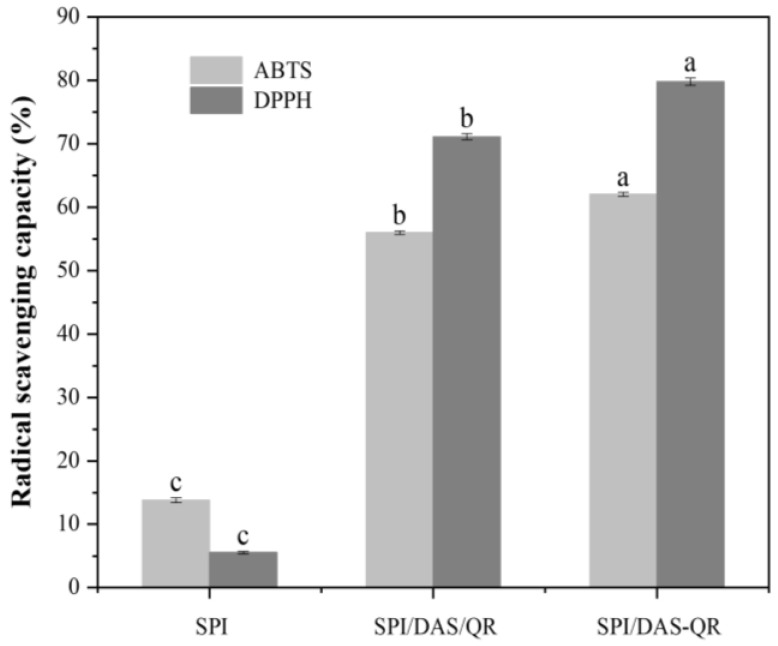
DPPH and ABTS radical scavenging activities of the SPI, SPI/DAS/QR, and SPI/DAS-QR films. a–c: different leters indicate significant differences among the films (*p* < 0.05).

Oxidation is a key factor that contributes to fruit spoilage and the degradation of nutritional components. Therefore, food packaging with antioxidant activity helps extend the shelf life of food. The antioxidant capacity of the SPI, SPI/DAS/QR, and SPI/DAS-QR films was determined by DPPH and ABTS radical scavenging assays. As shown in Figure 10, the SPI film exhibited weak antioxidant activity (DPPH: 5.5%; ABTS: 13.8%). The incorporation of DAS/QR and DAS-QR with SPI significantly improved the antioxidant activity. The DPPH radical scavenging activity was 71.4% and 79.8% for the SPI/DAS/QR and SPI/DAS-QR films, respectively, and the ABTS radical scavenging activity was 56.0% and 62.1%, respectively. The enhancement in the antioxidant capacity of the composite films was attributed to the QR moiety [39,40]. Notably, the antioxidant activity of SPI/DAS-QR was higher than that of SPI/DAS/QR. This may be because the stability of QR was improved by conjugating it with DAS, which resulted in the exertion of sustained and stable antioxidant activity [41].

### 3.9. Antibacterial Properties of the Films

**Figure 11:**
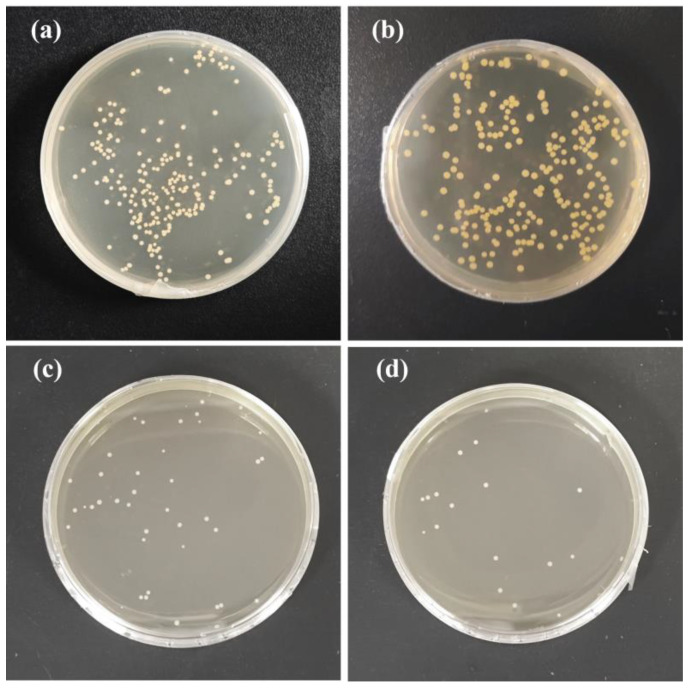
The inhibitory activity of (**a**) CK, (**b**) SPI, (**c**) SPI/DAS/QR, and (**d**) SPI/DAS-QR films against *S. aureus*.

Antibacterial capacity is another important indicator for active food packaging. As shown in Figure 11, the SPI film did not show antibacterial activity against *S. aureus*, while both SPI/DAS/QR and SPI/DAS-QR displayed significant antibacterial activity against *S. aureus*. SPI/DAS/QR and SPI/DAS-QR films exhibited inhibition rates of 83.4% and 92.7%, respectively. The improvement in the antibacterial capacity of the composite films was attributed to the QR moiety [39]. Notably, the antibacterial activity of SPI/DAS-QR was higher than that of SPI/DAS/QR. This may be because the stability of QR was improved by conjugating it with DAS, which resulted in the exertion of sustained and stable antibacterial activity [41]. In contrast, none of the films showed inhibitory activity against the Gram-negative bacteria *E. coli*, which may be attributed to its multi-layered cell wall structure.

### 3.10. Fresh-Cut Potato Preservation Application

**Figure 12:**
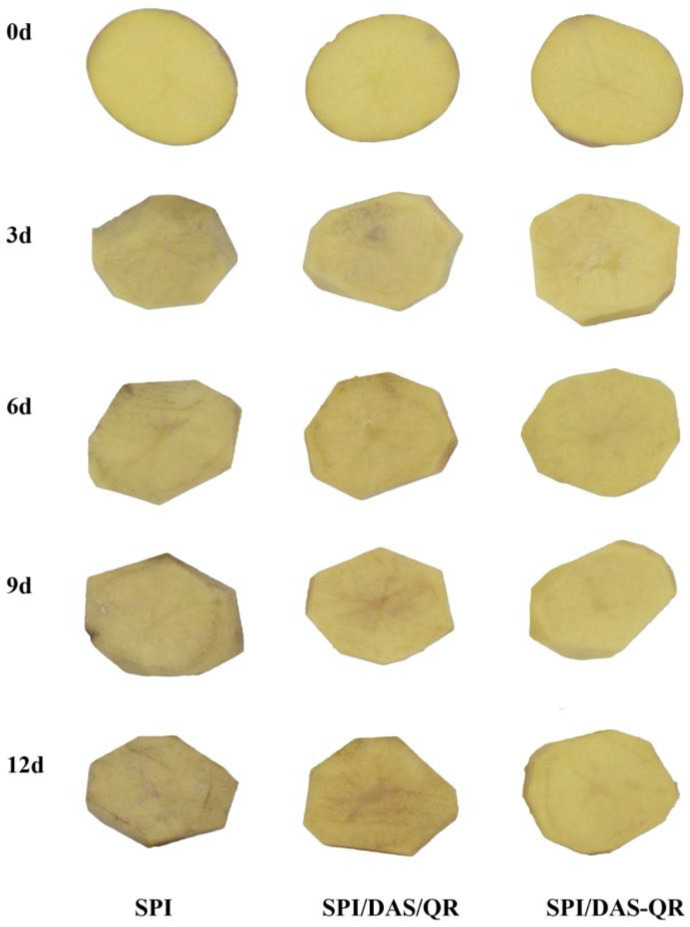
Appearance of the fresh-cut potatoes in the SPI, SPI/DAS/QR, and SPI/DAS-QR groups.

**Figure 13:**
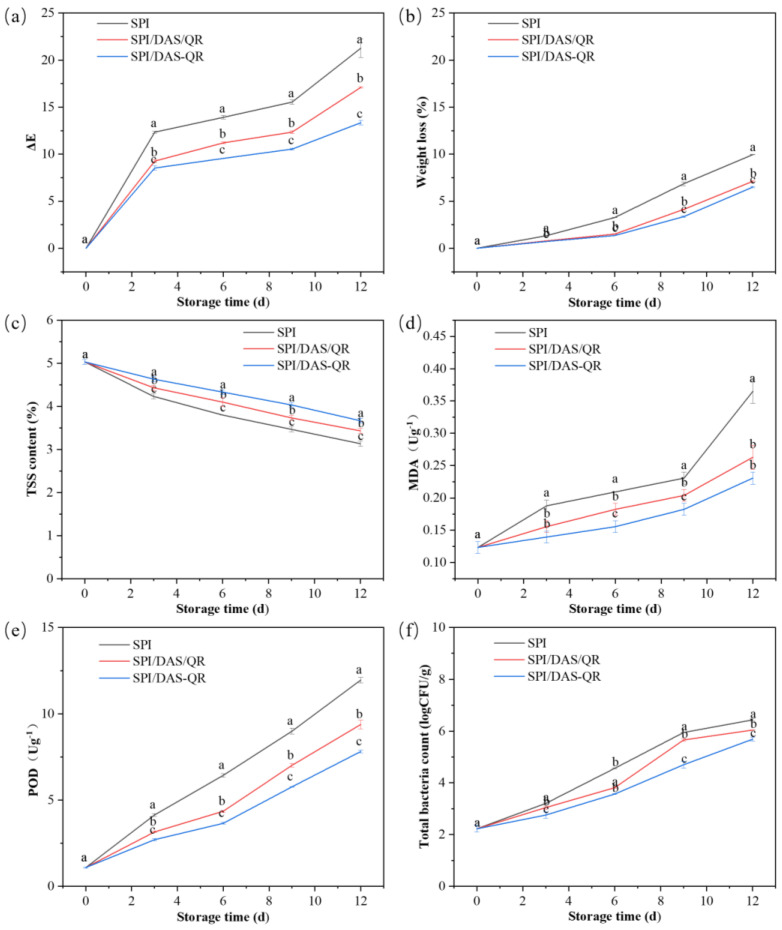
Color changes (**a**), weight loss (**b**), TSS content (**c**), MDA content (**d**), POD activity, (**e**) and total colony count (**f**) of the fresh-cut potatoes in the SPI, SPI/DAS/QR, and SPI/DAS-QR groups. a–c: different leters indicate significant differences among the films (*p* < 0.05).

Fresh-cut products are prone to oxidation and microbial contamination, leading to deterioration in appearance and texture, a loss of nutrients, and thus an affected consumption. The active packaging application was expected to maintain quality and decrease bacteria. As shown in Figure 12, the fresh-cut potatoes in the SPI, SPI/DAS/QR, and SPI/DAS-QR groups exhibited bright appearances at the beginning of the storage period. After the 12 d storage, the fresh-cut potatoes in the SPI group were more browned than those in the SPI/DAS/QR and SPI/DAS-QR groups. Notably, the samples in the SPI/DAS-QR group exhibited a brighter appearance than those in the SPI/DAS/QR group. The determined ΔE values could support this finding (Figure 13a). The results indicate that the SPI/DAS-QR coating film was helpful for maintaining the appearance of fresh-cut potatoes.

Figure 13b shows the effect of the SPI, SPI/DAS/QR, and SPI/DAS-QR coating films on the weight loss of the fresh-cut potatoes. SPI/DAS/QR and SPI/DAS-QR exhibited lower weight loss rates during the storage compared with the SPI group. The reason may be that the denser structures of SPI/DAS/QR and SPI/DAS-QR exhibited higher moisture-blocking capacity than that of SPI [42]. As shown in Figure 13c, the TSS content of the fresh-cut potatoes in all three groups decreased during the storage, which was caused by respiration that led to the consumption of carbohydrates. The SPI/DAS-QR coating film was more effective in delaying the decrease in the TSS content of the fresh-cut potatoes than the SPI and SPI /DAS/QR coating films. This may be due to its compact structure, endowing a high gas-blocking capacity that effectively reduced the respiration of the fresh-cut potatoes. MDA is an important indicator of plant senescence [43]. As shown in Figure 13d, the MDA content of fresh-cut potatoes in all three groups increased during the storage. The SPI/DAS-QR group exhibited the lowest MDA content compared with the SPI and SPI /DAS/QR groups. POD is one of the most important oxidoreductases in plants and an important enzyme involved in enzymatic browning [44]. Figure 13e displays the trend of POD activity, which was similar to that of MDA content. Overall, SPI/DAS-QR could effectively decrease the MDA content and the POD activity of fresh-cut potatoes. The reason may be that the dense SPI/DAS-QR coating film reduced the oxygen exchange and blocked the light, as well as its antioxidant activity. Figure 13f shows the effect of the SPI, SPI/DAS/QR, and SPI/DAS-QR coating films on the total colony count of the fresh-cut potatoes. The total colony count of all three groups increased during the storage. The SPI/DAS-QR group exhibited the lowest total colony count compared with the SPI and SPI /DAS/QR groups. This result was consistent with the result of the antibacterial assay. Notably, the total colony count of the fresh-cut potatoes in the SPI/DAS-QR group was less than 5.0 log CFU/g in the first 9 d of the storage period, which is within the acceptable microbiological limit of food.

## 4. Conclusions

In this study, SPI films incorporating DAS-QR and DAS/QR were developed, respectively. The light-blocking capacity, thermal stability, hydrophobicity, TS, EB, and antioxidant and antibacterial abilities of SPI film were improved by DAS-QR and DAS/QR. Notably, the SPI film incorporated with DAS-QR exhibited better performance than that with DAS/QR in terms of hydrophobicity, and antioxidant and antibacterial abilities. The composite coating film SPI/DAS-QR was more effective than SPI/DAS/QR in maintaining appearance quality, delaying the loss of weight and TSS, postponing MDA accumulation, and decreasing POD activity and microbial contamination in fresh-cut potatoes. The results suggest that the SPI/DAS-QR coating film possesses a good preservation effect on fresh-cut products. Therefore, the multifunctional coating film can be used as an active packaging material for fresh-cut product preservation.

## Figures and Tables

**Table 1:** Color of the SPI, SPI/DAS/QR, and SPI/DAS-QR films.

Film	L	a	b	ΔE
SPI	91.47 ± 0.53 ^a^	0.09 ± 0.00 ^c^	0.72 ± 0.04 ^c^	7.39 ± 0.05 ^c^
SPI/DAS/QR	84.15 ± 1.16 ^b^	4.72 ± 0.40 ^b^	37.82 ± 0.53 ^a^	45.44 ± 0.29 ^a^
SPI/DAS-QR	83.03 ± 0.30 ^b^	7.01 ± 0.81 ^a^	27.45 ± 0.09 ^b^	36.20 ± 0.32 ^b^

Data are presented as mean ± standard deviation (SD). Distinct superscript letters (a–c) within a given column denote statistically significant distinctions among the films (*p* < 0.05).

## Data Availability

The data presented in this study are available on request.

## Abbreviations

Soy protein isolate, SPI; dialdehyde starch, DAS; tensile strength, TS; chitosan, CS; quercetin, QR; *Listeria monocytogenes*, *L. monocytogenes*; *Escherichia coli*, *E. coli*; *Staphylococcus aureus*, *S. aureus*; 2, 2-diphenyl-1-picrylhydrazyl, DPPH; 2, 2′-azino-bis(3-ethylbenzothiazoline-6-sulfonic acid), ABTS; malondialdehyde, MDA; peroxidase, POD; deep eutectic solvent, DES; film-forming solution, FFS; relative humidity, RH; thermal gravimetric analysis, TGA; water contact angle, WCA; tensile strength, TS; elongation at break, EB; total soluble solids, TSS.
